# Supplementary material for: End Sequence Analysis Toolkit (ESAT) expands the extractable information from single-cell RNA-seq data
Source: Genome Res. 2016 Oct;26(10):1397–410. doi: 10.1101/gr.207902.116 (PMC5052061; doi:10.1101/gr.207902.116)
Supplement: Supplemental Material [file supp_gr.207902.116_Supp_Materials.tar.gz › ESAT-esat_v0.1/doc/html/classbroad_1_1pda_1_1seq_1_1graph_1_1_chromosome_with_bubbles_j_graph_t.html]

ESAT: broad.pda.seq.graph.ChromosomeWithBubblesJGraphT Class Reference


ESAT


- Main Page
- Related Pages
- Packages
- Classes
- Files

- Class List
- Class Index
- Class Hierarchy
- Class Members

All Classes Namespaces Files Functions Variables Enumerator Pages

- broad
- pda
- seq
- graph
- ChromosomeWithBubblesJGraphT

Classes |
Public Member Functions |
List of all members

broad.pda.seq.graph.ChromosomeWithBubblesJGraphT Class Reference

Inheritance diagram for broad.pda.seq.graph.ChromosomeWithBubblesJGraphT:

[legend]

Collaboration diagram for broad.pda.seq.graph.ChromosomeWithBubblesJGraphT:

[legend]

|  |  |
| --- | --- |
| Classes | |
| class | **BubbleEdge** |
|  | |
| enum | EdgeSourceType |
|  | |
| class | **ForwardAndBackIterator** |
|  | |
| class | **MinReadFilter** |
|  | |
| class | **RegionReadFilter** |
|  | |
| class | **ReverseWindowIterator** |
|  | |
| class | **WindowIterator** |
|  | |

|  |  |
| --- | --- |
| Public Member Functions | |
|  | ChromosomeWithBubblesJGraphT (String name) |
|  | |
|  | ChromosomeWithBubblesJGraphT (String name, int start, int end) |
|  | |
|  | ChromosomeWithBubblesJGraphT (String name, Collection<?extends Annotation > vertices, Collection<?extends Annotation > edges, Map<?extends Annotation, Double > edgeCounts, Map<?extends Annotation, Double > nodeCounts, double lambda, double numMarkers, double numReads, int minSplices) throws IllegalArgumentException |
|  | |
|  | ChromosomeWithBubblesJGraphT (String name, Collection<?extends Annotation > vertices, Collection<?extends Annotation > edges) throws IllegalArgumentException |
|  | |
|  | ChromosomeWithBubblesJGraphT (String name, Map<?extends Annotation, Double > nodes, Map< BubbleEdge, Double > edges, double lambda, double numMarkers, double numReads, double minEdgeSupport) |
|  | |
|  | ChromosomeWithBubblesJGraphT (String name, Collection<?extends Annotation > nodes, Collection<?extends Annotation > edges, double lambda, double numMarkers, double numReads, int minSplices) throws IllegalArgumentException |
|  | |
| boolean | addEdge (Annotation v1, Annotation v2, BubbleEdge e) |
|  | |
| boolean | addNewEdge (BubbleEdge e, Annotation v1, Annotation v2) |
|  | |
| boolean | addVertex (Annotation v) |
|  | |
| Collection< Gene > | getGenePaths (double alpha) |
|  | |
| Collection< Gene > | getGenePaths (double alpha, double minSpliceFrequency) |
|  | |
| Collection< Path > | getAllPaths () |
|  | |
| Collection< Path > | getAllPaths (double minSpliceFrequency) |
|  | |
| Collection< Gene > | getOrphanNodes () |
|  | |
| Collection< Gene > | getOrphanNodes (Alignments region) |
|  | |
| Collection< Path > | getOrphanPaths () |
|  | |
| Collection< Path > | getPaths (double alpha) |
|  | |
| Collection< Path > | getPaths (double alpha, double minSpliceFrequency) |
|  | |
| Set< BubbleEdge > | getEdges (Collection< Annotation > vertices) |
|  | |
| Collection< Annotation > | findReachableTargetVertices (Annotation source, Collection< Annotation > alreadyTraversed, Collection< Annotation > sinkVertices, Collection< Annotation > reachableSinks, int counter, DirectedGraph< Annotation, BubbleEdge > g) |
|  | |
| List< Path > | getPaths (Annotation startNode, Annotation endNode, double alpha, double minSpliceFreq, DirectedGraph< Annotation, BubbleEdge > g) |
|  | |
| List< Path > | getPaths (Annotation startNode, Annotation endNode, double alpha, Collection< EdgeSourceType > validEdgeTypes, double minSpliceFreq, DirectedGraph< Annotation, BubbleEdge > g) |
|  | |
| boolean | checkOrientation (Collection< BubbleEdge > edges) |
|  | |
| Annotation | getOppositeVertex (Annotation v, BubbleEdge e) |
|  | |
| Collection< Gene > | getPaths (int start, int end) |
|  | |
| Collection< Gene > | getPaths (int start, int end, boolean forward) |
|  | |
| Collection< Gene > | getPaths (int start, int end, double alpha, boolean forward) |
|  | |
| Collection< Gene > | getPaths (int start, int end, double alpha, Collection< EdgeSourceType > validTypes) |
|  | |
| Collection< Gene > | getBackwardPaths (int start, int end, double alpha, Collection< EdgeSourceType > validTypes) |
|  | |
| Collection< BubbleEdge > | getBackwardEdges (Annotation vertex, double alpha, Collection< EdgeSourceType > validTypes) |
|  | |
| void | setSpliceWeight (double spliceWeight) |
|  | |
| Collection< BubbleEdge > | getIncidentEdges (Annotation vertex) |
|  | |
| Collection< Annotation > | getSourceVertices (Collection< EdgeSourceType > validTypes, DirectedGraph< Annotation, BubbleEdge > g) |
|  | |
| Collection< Annotation > | getSinkVertices (Collection< EdgeSourceType > validTypes, DirectedGraph< Annotation, BubbleEdge > g) |
|  | |
| Collection< BubbleEdge > | getForwardEdges (Annotation vertex, double alpha, Collection< EdgeSourceType > validTypes) |
|  | |
| Collection< BubbleEdge > | getValidOutgoingEdges (Annotation vertex, double alpha, Collection< EdgeSourceType > validTypes, DirectedGraph< Annotation, BubbleEdge > g) |
|  | |
| int | inOrientedDegree (Annotation v, Collection< EdgeSourceType > validTypes, DirectedGraph< Annotation, BubbleEdge > g) |
|  | |
| int | outOrientedDegree (Annotation v, Collection< EdgeSourceType > validTypes, DirectedGraph< Annotation, BubbleEdge > g) |
|  | |
| boolean | isSpanningGap (int start, int end) |
|  | |
| boolean | isGap (int start, int end) |
|  | |
| boolean | isInAllGaps (int start, int end) |
|  | |
| void | removeSpanningGapNodes () |
|  | |
| boolean | removeVertex (Annotation vertex) |
|  | |
| boolean | removeEdge (BubbleEdge e) |
|  | |
| Collection< BubbleEdge > | getOutOrUnorientedEdges (Annotation v) |
|  | |
| Collection< BubbleEdge > | getInOrUnorientedEdges (Annotation v) |
|  | |
| void | addEdgeAddingMissingVertices (Annotation intron, float count) |
|  | |
| Annotation | findEdge (int start, int end) |
|  | |
| List< Annotation > | getOverlappingVertices (int start, int end) |
|  | |
| BubbleEdge | createEdge (Alignments connection, Annotation v1, Annotation v2, Integer count, EdgeSourceType type) |
|  | |
| VertexPair< Annotation > | getNodePair (BubbleEdge edge) |
|  | |
| WindowIterator | iterator (int windowSize, int start, int overlap) |
|  | |
| WindowIterator | iterator (int windowSize, int start, int overlap, boolean skipGapSpanningWindows) |
|  | |
| WindowIterator | iterator (int windowSize, int start) |
|  | |
| double | getLocalRate (Annotation exon) |
|  | |
| double | getLambda () |
|  | |
| int | getRelativeGenomeLength () |
|  | |
| double | getNumberOfMarkers () |
|  | |
| double | getNumberOfReads () |
|  | |
| String | getName () |
|  | |
| double | getRPKMConstant () |
|  | |
| Map< Annotation, Double > | getLocalRate () |
|  | |
| int | getEnd () |
|  | |
| int | getStart () |
|  | |
| void | setStart (int start) |
|  | |
| void | setEnd (int end) |
|  | |
| double | getSpliceWeight () |
|  | |
| int | getChromosomeLength () |
|  | |
| void | setLocalRate (Collection< Path > paths) |
|  | |
| void | setLocalRate (Map< Annotation, Double > localRateMap) |
|  | |
| void | setRPKMConstant (double num) |
|  | |
| void | setExonScores (Annotation node, Double score) |
|  | |
| void | setChromosomeLength (int chromosomeLength) |
|  | |
| void | writeGraph (String save, boolean setUpShortExonLabels) throws IOException |
|  | |
| void | writeGraph (String save) throws IOException |
|  | |
| void | writeGraph (String save, Alignments region) throws IOException |
|  | |
| void | writeGraph (String save, Alignments region, boolean setUpShortExonLabels) throws IOException |
|  | |
| void | writeGraph (String save, Alignments region, boolean setUpShortExonLabels, String graphName) throws IOException |
|  | |

## Detailed Description

Implements jung.Graph for Dijkstra (FOR NOW ONLY)

## Constructor & Destructor Documentation

|  |  |  |  |  |  |
| --- | --- | --- | --- | --- | --- |
| broad.pda.seq.graph.ChromosomeWithBubblesJGraphT.ChromosomeWithBubblesJGraphT | ( | String | *name* | ) |  |

CONSTRUCTORS TODO: A constructor that instantiates the class with a graph. Application: subgraph

|  |  |  |  |
| --- | --- | --- | --- |
| broad.pda.seq.graph.ChromosomeWithBubblesJGraphT.ChromosomeWithBubblesJGraphT | ( | String | *name*, |
|  |  | int | *start*, |
|  |  | int | *end* |
|  | ) |  |  |

|  |  |  |  |
| --- | --- | --- | --- |
| broad.pda.seq.graph.ChromosomeWithBubblesJGraphT.ChromosomeWithBubblesJGraphT | ( | String | *name*, |
|  |  | Collection<?extends Annotation > | *vertices*, |
|  |  | Collection<?extends Annotation > | *edges*, |
|  |  | Map<?extends Annotation, Double > | *edgeCounts*, |
|  |  | Map<?extends Annotation, Double > | *nodeCounts*, |
|  |  | double | *lambda*, |
|  |  | double | *numMarkers*, |
|  |  | double | *numReads*, |
|  |  | int | *minSplices* |
|  | ) |  | throws IllegalArgumentException |

Constructs a chromosome with bubbles with a given set of nodes and connections between them. The graph will keep track of how many times a given edge has been added. Nodes in this case are though as exons of putative transcripts.

Parameters
:   |  |  |
    | --- | --- |
    | name | - Name of the chromosome. |
    | vertices | Collection of nodes, note than duplicate nodes will be ignored |
    | edges | Collection of edges connecting nodes. We assume a close-open( [start, end) ) specification of the annotation, thus edge connects two nodes if the start of the edge is node1.getEnd() and the end of the edge is node2.getStart() - 1. **Edges must be sorted**. |
    | edgeCounts | Map of edges to their counts |
    | nodeCounts | Map of vertices to their counts |
    | lambda |  |
    | numMarkers |  |
    | numReads |  |
    | minSplices |  |

Exceptions
:   |  |  |
    | --- | --- |
    | IllegalArgumentException | if a node or and edge are on a different chromsome than the chromosomeWithBubbles instance. |

Here is the call graph for this function:

|  |  |  |  |
| --- | --- | --- | --- |
| broad.pda.seq.graph.ChromosomeWithBubblesJGraphT.ChromosomeWithBubblesJGraphT | ( | String | *name*, |
|  |  | Collection<?extends Annotation > | *vertices*, |
|  |  | Collection<?extends Annotation > | *edges* |
|  | ) |  | throws IllegalArgumentException |

Constructs a chromosome with bubbles with a given set of nodes and connections between them. The graph will keep track of how many times a given edge has been added. Nodes in this case are though as exons of putative transcripts.

Parameters
:   |  |  |
    | --- | --- |
    | name | - Name of the chromosome. |
    | vertices | Collection of nodes, note than duplicate nodes will be ignored |
    | edges | Collection of edges connecting nodes. We assume a close-open( [start, end) ) specification of the annotation, thus edge connects two nodes if the start of the edge is node1.getEnd() and the end of the edge is node2.getStart() - 1. **Edges must be sorted**. |

Exceptions
:   |  |  |
    | --- | --- |
    | IllegalArgumentException | if a node or and edge are on a different chromsome than the chromosomeWithBubbles instance. |

|  |  |  |  |
| --- | --- | --- | --- |
| broad.pda.seq.graph.ChromosomeWithBubblesJGraphT.ChromosomeWithBubblesJGraphT | ( | String | *name*, |
|  |  | Map<?extends Annotation, Double > | *nodes*, |
|  |  | Map< BubbleEdge, Double > | *edges*, |
|  |  | double | *lambda*, |
|  |  | double | *numMarkers*, |
|  |  | double | *numReads*, |
|  |  | double | *minEdgeSupport* |
|  | ) |  |  |

Constructs a chromosome with bubbles with a given set of nodes, edges.

Parameters
:   |  |  |
    | --- | --- |
    | name | Name of the chromosome. |
    | nodes | Collection of nodes, note than duplicate nodes will be ignored |
    | edges | Collection of edges connecting nodes. We assume a close-open( [start, end) ) specification of the annotation, thus edge connects two nodes if the start of the edge is node1.getEnd() and the end of the edge is node2.getStart() - 1. **Edges must be sorted**. |
    | lambda |  |
    | numMarkers |  |
    | numReads |  |
    | minEdgeSupport |  |

Here is the call graph for this function:

|  |  |  |  |
| --- | --- | --- | --- |
| broad.pda.seq.graph.ChromosomeWithBubblesJGraphT.ChromosomeWithBubblesJGraphT | ( | String | *name*, |
|  |  | Collection<?extends Annotation > | *nodes*, |
|  |  | Collection<?extends Annotation > | *edges*, |
|  |  | double | *lambda*, |
|  |  | double | *numMarkers*, |
|  |  | double | *numReads*, |
|  |  | int | *minSplices* |
|  | ) |  | throws IllegalArgumentException |

//??? This constructor is not used. And does not return the correct paths

Parameters
:   |  |  |
    | --- | --- |
    | name |  |
    | nodes |  |
    | edges |  |
    | fromBE | //??? //NOT USED |
    | lambda |  |
    | numMarkers |  |
    | numReads | ??? This will always return a Null Pointer Exception |
    | name |  |
    | nodes |  |
    | edges |  |
    | lambda |  |
    | numMarkers |  |
    | numReads |  |
    | minSplices |  |

Exceptions
:   |  |  |
    | --- | --- |
    | IllegalArgumentException |  |

## Member Function Documentation

|  |  |  |  |
| --- | --- | --- | --- |
| boolean broad.pda.seq.graph.ChromosomeWithBubblesJGraphT.addEdge | ( | Annotation | *v1*, |
|  |  | Annotation | *v2*, |
|  |  | BubbleEdge | *e* |
|  | ) |  |  |

Adds an edge e between vertices v1 and v2

Parameters
:   |  |  |
    | --- | --- |
    | e |  |
    | v1 |  |
    | v2 |  |

Returns

Here is the call graph for this function:

Here is the caller graph for this function:

|  |  |  |  |
| --- | --- | --- | --- |
| void broad.pda.seq.graph.ChromosomeWithBubblesJGraphT.addEdgeAddingMissingVertices | ( | Annotation | *intron*, |
|  |  | float | *count* |
|  | ) |  |  |

Returns a collection of edges of the allowed edge types that originate at the specified vertex (if oriented) or have the specified vertex as an end point

Parameters
:   |  |  |
    | --- | --- |
    | v | Specified vertex |

Returns
:   collection of edges Creates bubbles that connect the splice junctions specified in the data model. Nodes in the graphs (bubbles) are single base pairs indicating the splice junction.

Parameters
:   |  |  |
    | --- | --- |
    | model |  |
    | minAligmentGap | Adds an edge to the graph, adding missing vertices to the graph. |
    | intron |  |
    | count |  |

Here is the call graph for this function:

|  |  |  |  |
| --- | --- | --- | --- |
| boolean broad.pda.seq.graph.ChromosomeWithBubblesJGraphT.addNewEdge | ( | BubbleEdge | *e*, |
|  |  | Annotation | *v1*, |
|  |  | Annotation | *v2* |
|  | ) |  |  |

Adds a new edge between v1 and v2

Parameters
:   |  |  |
    | --- | --- |
    | e |  |
    | v1 |  |
    | v2 |  |

Returns

Here is the call graph for this function:

|  |  |  |  |  |  |
| --- | --- | --- | --- | --- | --- |
| boolean broad.pda.seq.graph.ChromosomeWithBubblesJGraphT.addVertex | ( | Annotation | *v* | ) |  |

Adds vertex v to the graph

Parameters
:   |  |  |
    | --- | --- |
    | v |  |

Here is the caller graph for this function:

|  |  |  |  |  |  |
| --- | --- | --- | --- | --- | --- |
| boolean broad.pda.seq.graph.ChromosomeWithBubblesJGraphT.checkOrientation | ( | Collection< BubbleEdge > | *edges* | ) |  |

|  |  |  |  |
| --- | --- | --- | --- |
| BubbleEdge broad.pda.seq.graph.ChromosomeWithBubblesJGraphT.createEdge | ( | Alignments | *connection*, |
|  |  | Annotation | *v1*, |
|  |  | Annotation | *v2*, |
|  |  | Integer | *count*, |
|  |  | EdgeSourceType | *type* |
|  | ) |  |  |

Creates an edge of type between v1 and v2 for connection alignment with weight

Parameters
:   |  |  |
    | --- | --- |
    | connection |  |
    | v1 |  |
    | v2 |  |
    | count |  |
    | type |  |

Returns

|  |  |  |  |
| --- | --- | --- | --- |
| Annotation broad.pda.seq.graph.ChromosomeWithBubblesJGraphT.findEdge | ( | int | *start*, |
|  |  | int | *end* |
|  | ) |  |  |

Returns edge between start and end

Parameters
:   |  |  |
    | --- | --- |
    | start |  |
    | end |  |

Returns

|  |  |  |  |
| --- | --- | --- | --- |
| Collection<Annotation> broad.pda.seq.graph.ChromosomeWithBubblesJGraphT.findReachableTargetVertices | ( | Annotation | *source*, |
|  |  | Collection< Annotation > | *alreadyTraversed*, |
|  |  | Collection< Annotation > | *sinkVertices*, |
|  |  | Collection< Annotation > | *reachableSinks*, |
|  |  | int | *counter*, |
|  |  | DirectedGraph< Annotation, BubbleEdge > | *g* |
|  | ) |  |  |

Here is the call graph for this function:

Here is the caller graph for this function:

|  |  |  |  |  |
| --- | --- | --- | --- | --- |
| Collection<Path> broad.pda.seq.graph.ChromosomeWithBubblesJGraphT.getAllPaths | ( |  | ) |  |

Returns the collection of Paths through the graph. Will call getAllPaths with 0 minimum splicing frequency.

Returns
:   collection of paths

|  |  |  |  |  |  |
| --- | --- | --- | --- | --- | --- |
| Collection<Path> broad.pda.seq.graph.ChromosomeWithBubblesJGraphT.getAllPaths | ( | double | *minSpliceFrequency* | ) |  |

Returns the collection of Paths through the graph with the minimum splice frequency

Parameters
:   |  |  |
    | --- | --- |
    | minSpliceFrequency | minimum splice frequency for the edges |

Returns

Here is the call graph for this function:

|  |  |  |  |
| --- | --- | --- | --- |
| Collection<BubbleEdge> broad.pda.seq.graph.ChromosomeWithBubblesJGraphT.getBackwardEdges | ( | Annotation | *vertex*, |
|  |  | double | *alpha*, |
|  |  | Collection< EdgeSourceType > | *validTypes* |
|  | ) |  |  |

Here is the call graph for this function:

Here is the caller graph for this function:

|  |  |  |  |
| --- | --- | --- | --- |
| Collection<Gene> broad.pda.seq.graph.ChromosomeWithBubblesJGraphT.getBackwardPaths | ( | int | *start*, |
|  |  | int | *end*, |
|  |  | double | *alpha*, |
|  |  | Collection< EdgeSourceType > | *validTypes* |
|  | ) |  |  |

Here is the call graph for this function:

Here is the caller graph for this function:

|  |  |  |  |  |
| --- | --- | --- | --- | --- |
| int broad.pda.seq.graph.ChromosomeWithBubblesJGraphT.getChromosomeLength | ( |  | ) |  |

|  |  |  |  |  |  |
| --- | --- | --- | --- | --- | --- |
| Set<BubbleEdge> broad.pda.seq.graph.ChromosomeWithBubblesJGraphT.getEdges | ( | Collection< Annotation > | *vertices* | ) |  |

Returns all edges connecting the specified vertices

Parameters
:   |  |  |
    | --- | --- |
    | vertices |  |

Returns

|  |  |  |  |  |
| --- | --- | --- | --- | --- |
| int broad.pda.seq.graph.ChromosomeWithBubblesJGraphT.getEnd | ( |  | ) |  |

Here is the caller graph for this function:

|  |  |  |  |
| --- | --- | --- | --- |
| Collection<BubbleEdge> broad.pda.seq.graph.ChromosomeWithBubblesJGraphT.getForwardEdges | ( | Annotation | *vertex*, |
|  |  | double | *alpha*, |
|  |  | Collection< EdgeSourceType > | *validTypes* |
|  | ) |  |  |

Here is the call graph for this function:

Here is the caller graph for this function:

|  |  |  |  |  |  |
| --- | --- | --- | --- | --- | --- |
| Collection<Gene> broad.pda.seq.graph.ChromosomeWithBubblesJGraphT.getGenePaths | ( | double | *alpha* | ) |  |

Returns a collection of paired end edges

Returns

|  |  |  |  |
| --- | --- | --- | --- |
| Collection<Gene> broad.pda.seq.graph.ChromosomeWithBubblesJGraphT.getGenePaths | ( | double | *alpha*, |
|  |  | double | *minSpliceFrequency* |
|  | ) |  |  |

Here is the call graph for this function:

|  |  |  |  |  |  |
| --- | --- | --- | --- | --- | --- |
| Collection<BubbleEdge> broad.pda.seq.graph.ChromosomeWithBubblesJGraphT.getIncidentEdges | ( | Annotation | *vertex* | ) |  |

Returns a collection of all incoming and outgoing edges of specified vertex

Parameters
:   |  |  |
    | --- | --- |
    | vertex |  |

Returns
:   collection of all edges for specified vertex

Here is the caller graph for this function:

|  |  |  |  |  |  |
| --- | --- | --- | --- | --- | --- |
| Collection<BubbleEdge> broad.pda.seq.graph.ChromosomeWithBubblesJGraphT.getInOrUnorientedEdges | ( | Annotation | *v* | ) |  |

Returns a collection of edges that terminate at the specified vertex (if oriented) or have the specified vertex as an end point

Parameters
:   |  |  |
    | --- | --- |
    | v | Specified vertex |

Returns
:   collection of edges

|  |  |  |  |  |
| --- | --- | --- | --- | --- |
| double broad.pda.seq.graph.ChromosomeWithBubblesJGraphT.getLambda | ( |  | ) |  |

Here is the caller graph for this function:

|  |  |  |  |  |  |
| --- | --- | --- | --- | --- | --- |
| double broad.pda.seq.graph.ChromosomeWithBubblesJGraphT.getLocalRate | ( | Annotation | *exon* | ) |  |

Here is the caller graph for this function:

|  |  |  |  |  |
| --- | --- | --- | --- | --- |
| Map<Annotation, Double> broad.pda.seq.graph.ChromosomeWithBubblesJGraphT.getLocalRate | ( |  | ) |  |

|  |  |  |  |  |
| --- | --- | --- | --- | --- |
| String broad.pda.seq.graph.ChromosomeWithBubblesJGraphT.getName | ( |  | ) |  |

Here is the caller graph for this function:

|  |  |  |  |  |  |
| --- | --- | --- | --- | --- | --- |
| VertexPair<Annotation> broad.pda.seq.graph.ChromosomeWithBubblesJGraphT.getNodePair | ( | BubbleEdge | *edge* | ) |  |

Returns the pair of vertices for a specified edge

Parameters
:   |  |  |
    | --- | --- |
    | edge |  |

Returns

Here is the caller graph for this function:

|  |  |  |  |  |
| --- | --- | --- | --- | --- |
| double broad.pda.seq.graph.ChromosomeWithBubblesJGraphT.getNumberOfMarkers | ( |  | ) |  |

|  |  |  |  |  |
| --- | --- | --- | --- | --- |
| double broad.pda.seq.graph.ChromosomeWithBubblesJGraphT.getNumberOfReads | ( |  | ) |  |

Here is the caller graph for this function:

|  |  |  |  |
| --- | --- | --- | --- |
| Annotation broad.pda.seq.graph.ChromosomeWithBubblesJGraphT.getOppositeVertex | ( | Annotation | *v*, |
|  |  | BubbleEdge | *e* |
|  | ) |  |  |

Returns the opposite vertex of the edge e from the vertex v

Parameters
:   |  |  |
    | --- | --- |
    | v |  |
    | e |  |

Returns

Here is the caller graph for this function:

|  |  |  |  |  |
| --- | --- | --- | --- | --- |
| Collection<Gene> broad.pda.seq.graph.ChromosomeWithBubblesJGraphT.getOrphanNodes | ( |  | ) |  |

Returns all orphan nodes in graph (Vertices without any edges orphan vertices)

Returns

Here is the call graph for this function:

|  |  |  |  |  |  |
| --- | --- | --- | --- | --- | --- |
| Collection<Gene> broad.pda.seq.graph.ChromosomeWithBubblesJGraphT.getOrphanNodes | ( | Alignments | *region* | ) |  |

Returns all orphan nodes in region (Vertices without any edges orphan vertices)

Parameters
:   |  |  |
    | --- | --- |
    | region |  |

Returns

Here is the call graph for this function:

|  |  |  |  |  |
| --- | --- | --- | --- | --- |
| Collection<Path> broad.pda.seq.graph.ChromosomeWithBubblesJGraphT.getOrphanPaths | ( |  | ) |  |

Returns all orphan paths in graph (self edges for orphan vertices)

Returns

Here is the call graph for this function:

Here is the caller graph for this function:

|  |  |  |  |  |  |
| --- | --- | --- | --- | --- | --- |
| Collection<BubbleEdge> broad.pda.seq.graph.ChromosomeWithBubblesJGraphT.getOutOrUnorientedEdges | ( | Annotation | *v* | ) |  |

Returns a collection of edges that are of the allowed edge types.

Parameters
:   |  |  |
    | --- | --- |
    | edgeList |  |

Returns
:   filtered edges Returns a collection of edges that originate at the specified vertex (if oriented) or have the specified vertex as an end point

Parameters
:   |  |  |
    | --- | --- |
    | v | Specified vertex |

Returns
:   collection of edges

|  |  |  |  |
| --- | --- | --- | --- |
| List<Annotation> broad.pda.seq.graph.ChromosomeWithBubblesJGraphT.getOverlappingVertices | ( | int | *start*, |
|  |  | int | *end* |
|  | ) |  |  |

Returns a list of all vertices overlapping region between start and end

Parameters
:   |  |  |
    | --- | --- |
    | start |  |
    | end |  |

Returns

|  |  |  |  |  |  |
| --- | --- | --- | --- | --- | --- |
| Collection<Path> broad.pda.seq.graph.ChromosomeWithBubblesJGraphT.getPaths | ( | double | *alpha* | ) |  |

Returns a collection of paths with alpha

Parameters
:   |  |  |
    | --- | --- |
    | alpha |  |

Returns

Here is the caller graph for this function:

|  |  |  |  |
| --- | --- | --- | --- |
| Collection<Path> broad.pda.seq.graph.ChromosomeWithBubblesJGraphT.getPaths | ( | double | *alpha*, |
|  |  | double | *minSpliceFrequency* |
|  | ) |  |  |

Returns a collection of paths with alpha and minimum spicing frequency

Parameters
:   |  |  |
    | --- | --- |
    | alpha |  |
    | minSpliceFrequency | minimum splicing frequency |

Returns

Here is the call graph for this function:

|  |  |  |  |
| --- | --- | --- | --- |
| List<Path> broad.pda.seq.graph.ChromosomeWithBubblesJGraphT.getPaths | ( | Annotation | *startNode*, |
|  |  | Annotation | *endNode*, |
|  |  | double | *alpha*, |
|  |  | double | *minSpliceFreq*, |
|  |  | DirectedGraph< Annotation, BubbleEdge > | *g* |
|  | ) |  |  |

Returns a collection of all paths starting from startNode with alpha and minimum splicing frequency

Parameters
:   |  |  |
    | --- | --- |
    | startNode |  |
    | alpha |  |
    | minSpliceFreq | minimum splicing frequency |

Returns

Here is the call graph for this function:

|  |  |  |  |
| --- | --- | --- | --- |
| List<Path> broad.pda.seq.graph.ChromosomeWithBubblesJGraphT.getPaths | ( | Annotation | *startNode*, |
|  |  | Annotation | *endNode*, |
|  |  | double | *alpha*, |
|  |  | Collection< EdgeSourceType > | *validEdgeTypes*, |
|  |  | double | *minSpliceFreq*, |
|  |  | DirectedGraph< Annotation, BubbleEdge > | *g* |
|  | ) |  |  |

Returns a collection of all paths starting from startNode having the specified valid edge types

Parameters
:   |  |  |
    | --- | --- |
    | startNode |  |
    | alpha |  |
    | validEdgeTypes |  |
    | minSpliceFreq | minimum splicing frequency |

Returns

Here is the call graph for this function:

|  |  |  |  |
| --- | --- | --- | --- |
| Collection<Gene> broad.pda.seq.graph.ChromosomeWithBubblesJGraphT.getPaths | ( | int | *start*, |
|  |  | int | *end* |
|  | ) |  |  |

DO WE NEED THIS???? Returns collection of genes between start and end

Parameters
:   |  |  |
    | --- | --- |
    | start |  |
    | end |  |

Returns

Here is the call graph for this function:

|  |  |  |  |
| --- | --- | --- | --- |
| Collection<Gene> broad.pda.seq.graph.ChromosomeWithBubblesJGraphT.getPaths | ( | int | *start*, |
|  |  | int | *end*, |
|  |  | boolean | *forward* |
|  | ) |  |  |

Here is the call graph for this function:

|  |  |  |  |
| --- | --- | --- | --- |
| Collection<Gene> broad.pda.seq.graph.ChromosomeWithBubblesJGraphT.getPaths | ( | int | *start*, |
|  |  | int | *end*, |
|  |  | double | *alpha*, |
|  |  | boolean | *forward* |
|  | ) |  |  |

Here is the call graph for this function:

|  |  |  |  |
| --- | --- | --- | --- |
| Collection<Gene> broad.pda.seq.graph.ChromosomeWithBubblesJGraphT.getPaths | ( | int | *start*, |
|  |  | int | *end*, |
|  |  | double | *alpha*, |
|  |  | Collection< EdgeSourceType > | *validTypes* |
|  | ) |  |  |

Here is the call graph for this function:

|  |  |  |  |  |
| --- | --- | --- | --- | --- |
| int broad.pda.seq.graph.ChromosomeWithBubblesJGraphT.getRelativeGenomeLength | ( |  | ) |  |

|  |  |  |  |  |
| --- | --- | --- | --- | --- |
| double broad.pda.seq.graph.ChromosomeWithBubblesJGraphT.getRPKMConstant | ( |  | ) |  |

|  |  |  |  |
| --- | --- | --- | --- |
| Collection<Annotation> broad.pda.seq.graph.ChromosomeWithBubblesJGraphT.getSinkVertices | ( | Collection< EdgeSourceType > | *validTypes*, |
|  |  | DirectedGraph< Annotation, BubbleEdge > | *g* |
|  | ) |  |  |

Returns a collection of all the possible target/end vertices for this graph.

Parameters
:   |  |  |
    | --- | --- |
    | validTypes | List of allowed edge types. |

Returns
:   All vertices having allowed edge types.

Here is the call graph for this function:

Here is the caller graph for this function:

|  |  |  |  |
| --- | --- | --- | --- |
| Collection<Annotation> broad.pda.seq.graph.ChromosomeWithBubblesJGraphT.getSourceVertices | ( | Collection< EdgeSourceType > | *validTypes*, |
|  |  | DirectedGraph< Annotation, BubbleEdge > | *g* |
|  | ) |  |  |

Returns a collection of all the possible source/start vertices for the specified graph.

Parameters
:   |  |  |
    | --- | --- |
    | validTypes | List of allowed edge types. |

Returns
:   All vertices having allowed edge types.

Here is the call graph for this function:

Here is the caller graph for this function:

|  |  |  |  |  |
| --- | --- | --- | --- | --- |
| double broad.pda.seq.graph.ChromosomeWithBubblesJGraphT.getSpliceWeight | ( |  | ) |  |

|  |  |  |  |  |
| --- | --- | --- | --- | --- |
| int broad.pda.seq.graph.ChromosomeWithBubblesJGraphT.getStart | ( |  | ) |  |

Here is the caller graph for this function:

|  |  |  |  |
| --- | --- | --- | --- |
| Collection<BubbleEdge> broad.pda.seq.graph.ChromosomeWithBubblesJGraphT.getValidOutgoingEdges | ( | Annotation | *vertex*, |
|  |  | double | *alpha*, |
|  |  | Collection< EdgeSourceType > | *validTypes*, |
|  |  | DirectedGraph< Annotation, BubbleEdge > | *g* |
|  | ) |  |  |

Here is the caller graph for this function:

|  |  |  |  |
| --- | --- | --- | --- |
| int broad.pda.seq.graph.ChromosomeWithBubblesJGraphT.inOrientedDegree | ( | Annotation | *v*, |
|  |  | Collection< EdgeSourceType > | *validTypes*, |
|  |  | DirectedGraph< Annotation, BubbleEdge > | *g* |
|  | ) |  |  |

Returns the in degree of the given vertex for a list of allowed edge types

Parameters
:   |  |  |
    | --- | --- |
    | v | specific vertex |
    | validTypes | Collections of allowed edge types |

Returns
:   in degree of given vertex

Here is the caller graph for this function:

|  |  |  |  |
| --- | --- | --- | --- |
| boolean broad.pda.seq.graph.ChromosomeWithBubblesJGraphT.isGap | ( | int | *start*, |
|  |  | int | *end* |
|  | ) |  |  |

Returns true if position starts in gap but goes past gap

Parameters
:   |  |  |
    | --- | --- |
    | start |  |
    | end |  |

Returns

|  |  |  |  |
| --- | --- | --- | --- |
| boolean broad.pda.seq.graph.ChromosomeWithBubblesJGraphT.isInAllGaps | ( | int | *start*, |
|  |  | int | *end* |
|  | ) |  |  |

Returns true if the start and end positions are all found in gaps (introns??)

Parameters
:   |  |  |
    | --- | --- |
    | start |  |
    | end |  |

Returns

Here is the caller graph for this function:

|  |  |  |  |
| --- | --- | --- | --- |
| boolean broad.pda.seq.graph.ChromosomeWithBubblesJGraphT.isSpanningGap | ( | int | *start*, |
|  |  | int | *end* |
|  | ) |  |  |

Returns true if this region spans an intron. Also, returns true if the position starts in gap but goes past gap.

Parameters
:   |  |  |
    | --- | --- |
    | start |  |
    | end |  |

Returns

Here is the call graph for this function:

Here is the caller graph for this function:

|  |  |  |  |
| --- | --- | --- | --- |
| WindowIterator broad.pda.seq.graph.ChromosomeWithBubblesJGraphT.iterator | ( | int | *windowSize*, |
|  |  | int | *start*, |
|  |  | int | *overlap* |
|  | ) |  |  |

|  |  |  |  |
| --- | --- | --- | --- |
| WindowIterator broad.pda.seq.graph.ChromosomeWithBubblesJGraphT.iterator | ( | int | *windowSize*, |
|  |  | int | *start*, |
|  |  | int | *overlap*, |
|  |  | boolean | *skipGapSpanningWindows* |
|  | ) |  |  |

|  |  |  |  |
| --- | --- | --- | --- |
| WindowIterator broad.pda.seq.graph.ChromosomeWithBubblesJGraphT.iterator | ( | int | *windowSize*, |
|  |  | int | *start* |
|  | ) |  |  |

|  |  |  |  |
| --- | --- | --- | --- |
| int broad.pda.seq.graph.ChromosomeWithBubblesJGraphT.outOrientedDegree | ( | Annotation | *v*, |
|  |  | Collection< EdgeSourceType > | *validTypes*, |
|  |  | DirectedGraph< Annotation, BubbleEdge > | *g* |
|  | ) |  |  |

Returns the out degree of the given vertex for a list of allowed edge types

Parameters
:   |  |  |
    | --- | --- |
    | v | specific vertex |
    | validTypes | Collections of allowed edge types |

Returns
:   out degree of given vertex

Here is the caller graph for this function:

|  |  |  |  |  |  |
| --- | --- | --- | --- | --- | --- |
| boolean broad.pda.seq.graph.ChromosomeWithBubblesJGraphT.removeEdge | ( | BubbleEdge | *e* | ) |  |

Removes edge from graph

Parameters
:   |  |  |
    | --- | --- |
    | e |  |

Returns

Here is the call graph for this function:

Here is the caller graph for this function:

|  |  |  |  |  |
| --- | --- | --- | --- | --- |
| void broad.pda.seq.graph.ChromosomeWithBubblesJGraphT.removeSpanningGapNodes | ( |  | ) |  |

Removes all nodes that span a gap

Here is the call graph for this function:

|  |  |  |  |  |  |
| --- | --- | --- | --- | --- | --- |
| boolean broad.pda.seq.graph.ChromosomeWithBubblesJGraphT.removeVertex | ( | Annotation | *vertex* | ) |  |

Removes vertex from graph

Parameters
:   |  |  |
    | --- | --- |
    | vertex |  |

Here is the call graph for this function:

Here is the caller graph for this function:

|  |  |  |  |  |  |
| --- | --- | --- | --- | --- | --- |
| void broad.pda.seq.graph.ChromosomeWithBubblesJGraphT.setChromosomeLength | ( | int | *chromosomeLength* | ) |  |

|  |  |  |  |  |  |
| --- | --- | --- | --- | --- | --- |
| void broad.pda.seq.graph.ChromosomeWithBubblesJGraphT.setEnd | ( | int | *end* | ) |  |

Sets the end of this graph

Parameters
:   |  |  |
    | --- | --- |
    | end |  |

|  |  |  |  |
| --- | --- | --- | --- |
| void broad.pda.seq.graph.ChromosomeWithBubblesJGraphT.setExonScores | ( | Annotation | *node*, |
|  |  | Double | *score* |
|  | ) |  |  |

|  |  |  |  |  |  |
| --- | --- | --- | --- | --- | --- |
| void broad.pda.seq.graph.ChromosomeWithBubblesJGraphT.setLocalRate | ( | Collection< Path > | *paths* | ) |  |

|  |  |  |  |  |  |
| --- | --- | --- | --- | --- | --- |
| void broad.pda.seq.graph.ChromosomeWithBubblesJGraphT.setLocalRate | ( | Map< Annotation, Double > | *localRateMap* | ) |  |

|  |  |  |  |  |  |
| --- | --- | --- | --- | --- | --- |
| void broad.pda.seq.graph.ChromosomeWithBubblesJGraphT.setRPKMConstant | ( | double | *num* | ) |  |

|  |  |  |  |  |  |
| --- | --- | --- | --- | --- | --- |
| void broad.pda.seq.graph.ChromosomeWithBubblesJGraphT.setSpliceWeight | ( | double | *spliceWeight* | ) |  |

Sets the splice weight for this graph

Parameters
:   |  |  |
    | --- | --- |
    | spliceWeight | Value for splice weight |

|  |  |  |  |  |  |
| --- | --- | --- | --- | --- | --- |
| void broad.pda.seq.graph.ChromosomeWithBubblesJGraphT.setStart | ( | int | *start* | ) |  |

Sets the start of this graph

Parameters
:   |  |  |
    | --- | --- |
    | start |  |

|  |  |  |  |
| --- | --- | --- | --- |
| void broad.pda.seq.graph.ChromosomeWithBubblesJGraphT.writeGraph | ( | String | *save*, |
|  |  | boolean | *setUpShortExonLabels* |
|  | ) |  | throws IOException |

Write the graph as a DOT format

Parameters
:   |  |  |
    | --- | --- |
    | save |  |
    | setUpShortExonLabels |  |

Exceptions
:   |  |  |
    | --- | --- |
    | IOException |  |

Here is the call graph for this function:

Here is the caller graph for this function:

|  |  |  |  |  |  |
| --- | --- | --- | --- | --- | --- |
| void broad.pda.seq.graph.ChromosomeWithBubblesJGraphT.writeGraph | ( | String | *save* | ) | throws IOException |

Here is the call graph for this function:

|  |  |  |  |
| --- | --- | --- | --- |
| void broad.pda.seq.graph.ChromosomeWithBubblesJGraphT.writeGraph | ( | String | *save*, |
|  |  | Alignments | *region* |
|  | ) |  | throws IOException |

Here is the call graph for this function:

|  |  |  |  |
| --- | --- | --- | --- |
| void broad.pda.seq.graph.ChromosomeWithBubblesJGraphT.writeGraph | ( | String | *save*, |
|  |  | Alignments | *region*, |
|  |  | boolean | *setUpShortExonLabels* |
|  | ) |  | throws IOException |

Here is the call graph for this function:

|  |  |  |  |
| --- | --- | --- | --- |
| void broad.pda.seq.graph.ChromosomeWithBubblesJGraphT.writeGraph | ( | String | *save*, |
|  |  | Alignments | *region*, |
|  |  | boolean | *setUpShortExonLabels*, |
|  |  | String | *graphName* |
|  | ) |  | throws IOException |

Writes the graph to a file

Parameters
:   |  |  |
    | --- | --- |
    | save |  |
    | region |  |
    | setUpShortExonLabels |  |
    | graphName |  |

Exceptions
:   |  |  |
    | --- | --- |
    | IOException |  |

Here is the call graph for this function:

---

The documentation for this class was generated from the following file:

- java/broad/pda/seq/graph/ChromosomeWithBubblesJGraphT.java


---

Generated on Fri Aug 15 2014 15:11:02 for ESAT by  

 1.8.7
